# Septal chondrocyte hypertrophy contributes to midface deformity in a mouse model of Apert syndrome

**DOI:** 10.1038/s41598-021-87260-5

**Published:** 2021-04-12

**Authors:** Bong-Soo Kim, Hye-Rim Shin, Hyun-Jung Kim, Heein Yoon, Young-Dan Cho, Kang-Young Choi, Je-Yong Choi, Woo-Jin Kim, Hyun-Mo Ryoo

**Affiliations:** 1 Department of Molecular Genetics and Dental Pharmacology, School of Dentistry and Dental Research Institute, Seoul National University, Seoul, South Korea; 2 Department of Periodontology, School of Dentistry and Dental Research Institute, Seoul National University, Seoul, South Korea; 3 Department of Plastic and Reconstructive Surgery, School of Medicine, Kyungpook National University, Daegu, South Korea; 4 Department of Biochemistry and Cell Biology, Cell and Matrix Research Institute, Skeletal Disease Analysis Center, Korea Mouse Phenotyping Center (KMPC), School of Medicine, Kyungpook National University, Daegu, South Korea

**Keywords:** Mechanisms of disease, Disease genetics, Bone development, Cartilage development

## Abstract

Midface hypoplasia is a major manifestation of Apert syndrome. However, the tissue component responsible for midface hypoplasia has not been elucidated. We studied mice with a chondrocyte-specific *Fgfr2*^*S252W*^ mutation (*Col2a1-cre; Fgfr2*^*S252W/*+^) to investigate the effect of cartilaginous components in midface hypoplasia of Apert syndrome. In *Col2a1-cre; Fgfr2*^*S252W/*+^ mice, skull shape was normal at birth, but hypoplastic phenotypes became evident with age. General dimensional changes of mutant mice were comparable with those of mice with mutations in *EIIa-cre; Fgfr2*^*S252W/*+^, a classic model of Apert syndrome in mice. *Col2a1-cre; Fgfr2*^*S252W/*+^ mice showed some unique facial phenotypes, such as elevated nasion, abnormal fusion of the suture between the premaxilla and the vomer, and decreased perpendicular plate of the ethmoid bone volume, which are related to the development of the nasal septal cartilage. Morphological and histological examination revealed that the presence of increased septal chondrocyte hypertrophy and abnormal thickening of nasal septum is causally related to midface deformities in nasal septum-associated structures. Our results suggest that careful examination and surgical correction of the nasal septal cartilage may improve the prognosis in the surgical treatment of midface hypoplasia and respiratory problems in patients with Apert syndrome.

## Introduction

Activating mutations of the fibroblast growth factor receptor 2 (*FGFR2*) gene are well known to cause syndromic craniosynostosis, including Apert, Crouzon, and Pfeiffer syndromes^1^. In the case of Apert syndrome, mostly Ser252Trp or Pro253Arg mutations cause loss of ligand–receptor specificity and hyperactivation of downstream signaling^2^. Syndromic craniosynostosis is often characterized by midface hypoplasia, elevated intracranial pressure and breathing difficulties^3,4^. Surgical maxillary advancement should be conducted to reduce respiratory distress and intracranial hypertension^5^. However, because of the unpredictable growth pattern of the midface, a surgical correction might be repeated later in life with multidisciplinary approaches^6^.

Nasal septum development is essential in the midface; it is responsible for sagittal and vertical maxillary growth^7,8^. Patients with craniosynostosis can present with a narrow nose, septum deviation, and associated breathing issues^6,9^. Additionally, since the nasal septal cartilage articulates with various facial bones^10^, and has various roles as a growth center in the midface^11^, an understanding of the intrinsic growth potential of the nasal septal cartilage is therefore critical for surgical correction of craniofacial defects.

We previously reported that premature obliteration of the facial suture was the main contributor to the development of midface hypoplasia and skull anomalies in a mouse model of Apert syndrome^12^. However, the intrinsic growth of facial bony or cartilaginous components beyond the craniofacial suture closure is still poorly understood. In this study, we carefully observed mice with the chondrocyte-specific *Fgfr2*^*S252*^^*W*^ mutation to examine the growth of facial cartilaginous tissues and its effect on midface hypoplasia.

## Results

### Chondrocyte-specific ***Fgfr2***^***S252W***^ mutation shows a progressive midface hypoplasia in the absence of premature craniofacial suture closure

The mouse model of Apert syndrome (*EIIa-cre; Fgfr2*^*S252W/*+^; referred to hereafter as “*EIIa-SW*”) demonstrated high rates of neonatal and postnatal lethality (Supporting Fig. 3). Mice with the chondrocyte-specific *Fgfr2*^*S252W*^ mutation (*Col2a1-cre; Fgfr2*^*S252W/*+^; referred to as “*Col2-SW*” hereafter) showed an improved survival rate, but postnatal lethality was still observed (Supporting Fig. 3). Because the mice with mature osteoblast-specific *Fgfr2*^*S252W*^ mutations (*Col1a1(2.3 kb)-cre; Fgfr2*^*S252W/*+^; referred to as “*Col1-SW”* hereafter) showed normal survival rate and skull morphology (Supporting Fig. 3 and 4), we ruled out this genotype in this study. These results indicated that the presence of the *Fgfr2*^*S252W*^ mutation in the cartilaginous tissues was more responsible for the early midfacial deformities than that in mature osteoblasts.

**Figure 1:**
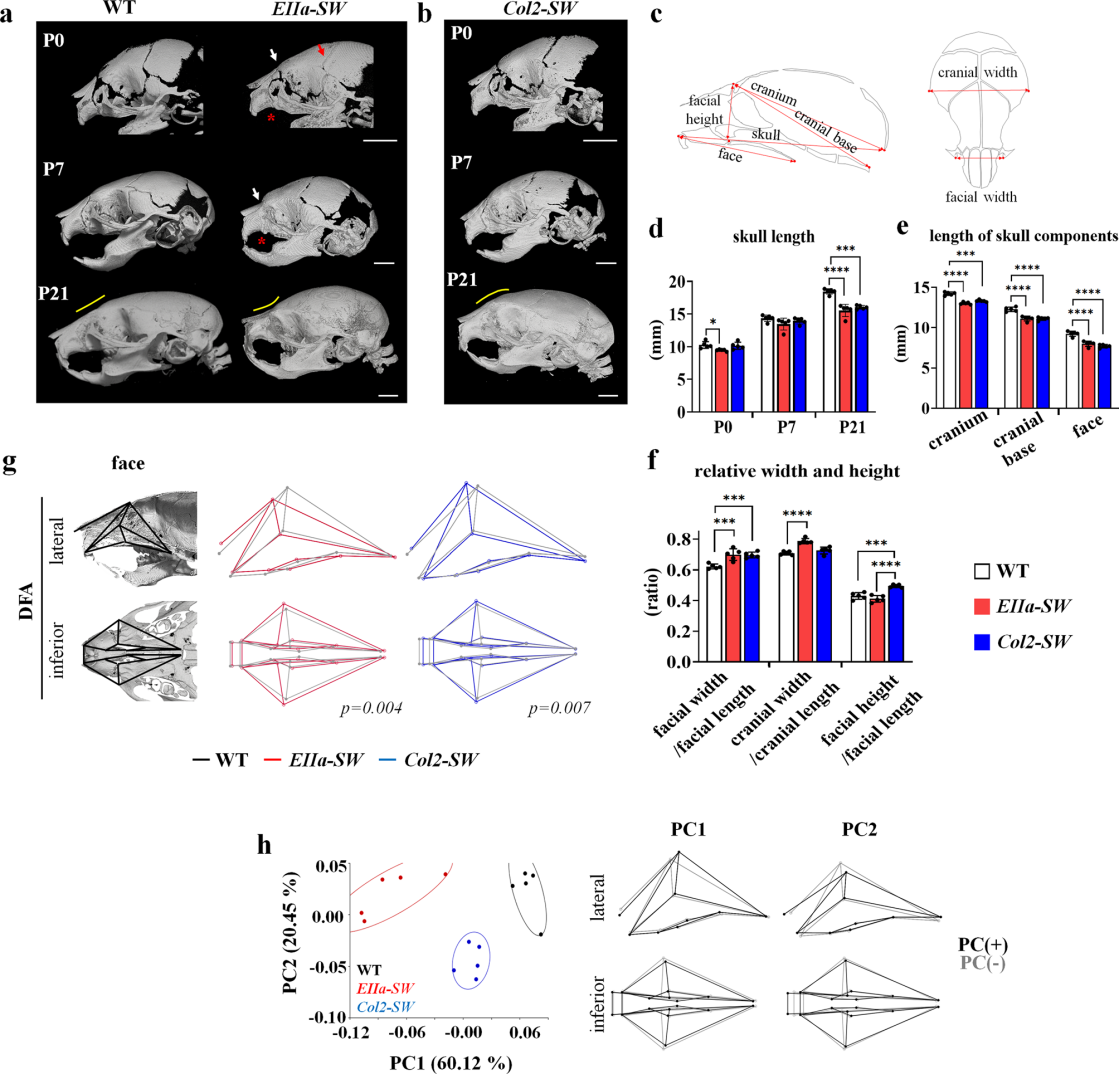
Chondrocyte-specific *Fgfr2*^*S252W*^ mutation shows progressive midface hypoplasia in the absence of premature craniofacial suture closure. (**a**) Micro-computed tomographic (CT) images of the skulls of the mouse model of Apert syndrome on postnatal days 0, 7, and 21 (P0, P7, and P21). The white and red arrows in the images of *EIIa-SW* mice indicate the fusion of nasofrontal and coronal sutures, respectively. The red asterisk indicates the fusion of the premaxillo–maxillary suture at P0 and P7. The yellow line indicates the curvature of the nasion. Abbreviations: pm, premaxilla; m, maxilla. Scale bar: 2 mm. (**b**) Micro-CT images of the skulls of *Col2-SW* mice at P0, P7, and P21. The yellow line indicates the curvature of the nasion. Scale bar: 2 mm. (**c**) Schematic of linear measurements of the craniofacial bones. (**d**) Skull lengths in each group at P0, P7, and P21. (n = 5, each stage) (**e**) Linear measurements of the cranium, the cranial base, and the face at P21 (n = 5). (**f**) The facial width and the height, and the cranial width in relation to their length at P21 (n = 5). (**g**) The facial shapes of the two groups were compared in discriminant function analysis (DFA). The first column indicates the analyzed facial region in the lateral and inferior views. The mean facial shape is displayed by wireframe images. The *p* values for permutation tests (1000 permutations) between the two groups are listed below of the wireframe images. (n = 5) (**h**) The variance in the facial shape was analyzed by principal components analysis (PCA). The percentage of total variance for each principal component is displayed on the axis. The shape changes are represented with wireframe images by the axis along the negative principal component axis (gray line) or positive principal component axis (black line) (n = 5). Values are presented as means ± standard deviations. *p ≤ 0.0332, **p ≤ 0.0021, ***p ≤ 0.0002, ****p ≤ 0.0001.

Micro-CT images of tissues of *EIIa-SW* mice on P0 showed early fusion of coronal, nasofrontal, and premaxillo–maxillary sutures (Fig. 1a). The premaxillo–maxillary suture, normally patent at P7, was obliterated in *EIIa-SW* mice at P0. On a postnatal day 21 (P21), *EIIa-SW* mice showed dramatically shortened skulls, severely sunken nasion and distorted noses. The *Col2-SW* mice showed normal craniofacial suture development (Fig. 1b, Supporting Fig. 5, and Supporting Table 4); however, they did show a progressive decrease in anteroposterior growth of the skull that was comparable with that of the *EIIa-SW* mice (Fig. 1c,d). The length of the skull components, the cranium, the cranial base, and the face displayed similar growth patterns, which resulted in significant shortening (Fig. 1e). Due to the significantly reduced skull size (Fig. 1d,e and Supporting Fig. 6), the relative width and height to the length were compared (Fig. 1f). The relative width of the face and the cranium were increased in the *EIIa-SW* mice (Fig. 1f). The *Col2-SW* mice also showed increased facial width, although the facial sutures were not closed prematurely. Especially, the facial height was increased with elevation of the nasion *Col2-SW* mice (Fig. 1f). Three-dimensional morphometric analyses based on the 23 craniofacial landmarks in mice at P21 (Supporting Fig. 3) demonstrated shortened face, sunken nasion, expanded cranium, and flexed cranial base in *EIIa-SW* mice (Supporting Fig. 7). *Col2-SW* mice also displayed shortening of anterior face, but the shape of cranial base and the cranial width were normal. In particular, the nasion was notably elevated as seen in the micro-CT images (Fig. 1g). The unique facial structure of *Col2-SW* mice, with elevated nasion and widened face, was completely distinguishable from those of wild type (WT) and *EIIa-SW* mice (Fig. 1h). These results suggest that when the mutation targets cartilage, the resulting dysmorphology is distinct from when it targets all tissues, suggesting a specific role for cartilage in the midfacial deformities.

### Facial deformities in *Col2-SW* mice are mainly developed in nasal septum-associated structure

**Figure 2:**
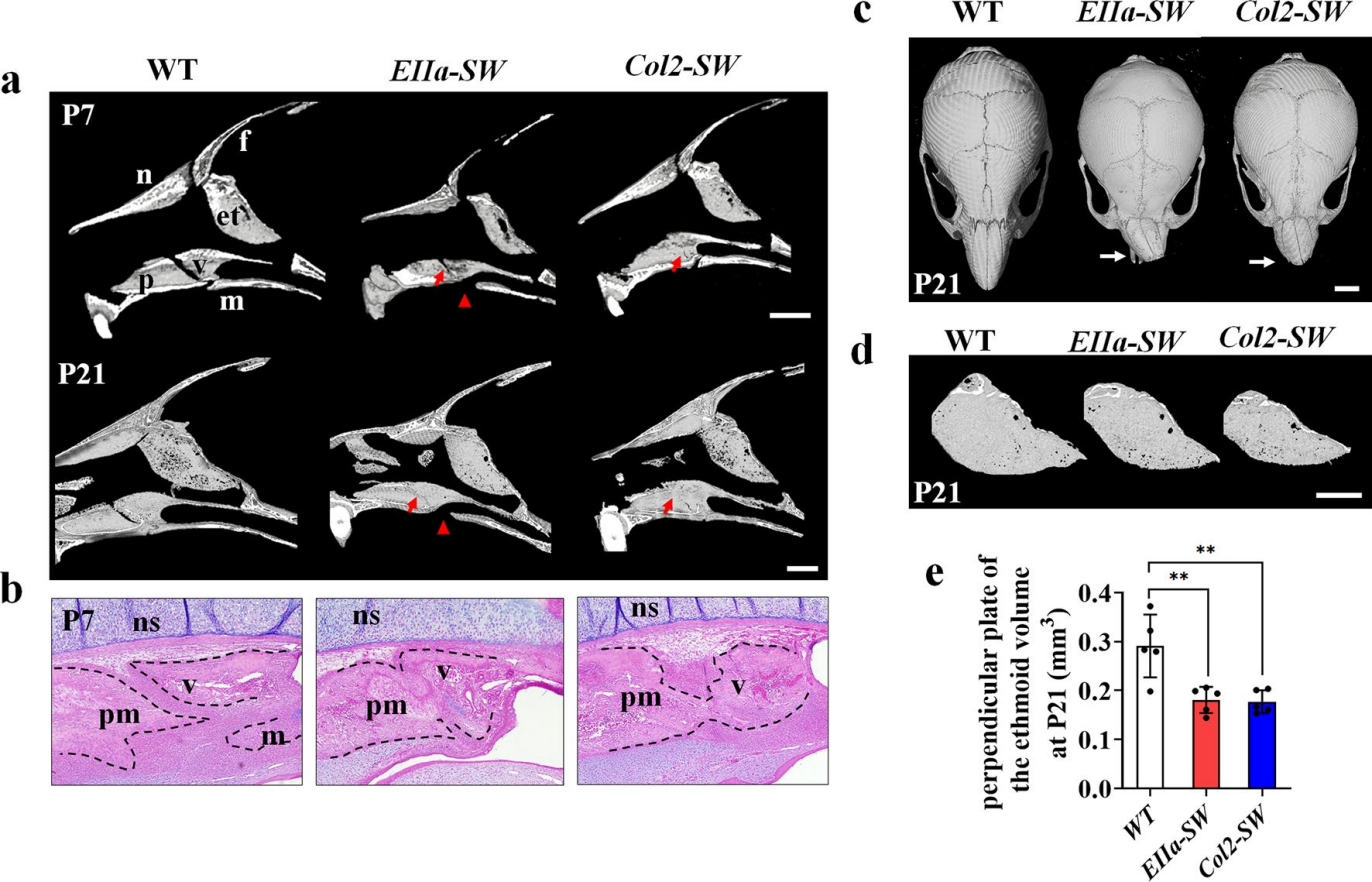
Facial deformities in *Col2-SW* mice are mainly developed in nasal septum-associated structure. (**a**) Micro-CT images of mouse skulls in the midsagittal plane at P7 and P21. The red arrows indicate the closure between the premaxilla and the vomer. The red arrowheads indicate palate perforation. Scale bar: 1 mm. (**b**) Hematoxylin, eosin, and Alcian blue staining of skulls of P7 mice in the midsagittal plane. The dotted line indicates the edge of each bone. Abbreviations: n, nasal bone; f, frontal bone; pm, premaxilla bone; v, vomer; et, ethmoid bone; m, maxilla; ns, nasal septum. Scale bar: 1 mm. (**c**) Superior view of the skull. The white arrows on the mutant mice indicate the deviation of the nasal bone. Scale bar: 2 mm. (**d**) Micro-CT images of the perpendicular plate of the ethmoid bone at P21. (**e**) Measurement of perpendicular plate of ethmoid bone volume. Scale bar: 1 mm. Values are presented as means ± standard deviations. *p ≤ 0.0332, **p ≤ 0.0021 (*n* = 5).

Because we found unique facial phenotypes in *Col2-SW* mice (Fig. 1), we focused on the intranasal and intrafacial regions to find possible causes of the craniofacial anomalies. We found that the vomero–premaxillary suture, which is normally patent even at P21, was abnormally fused in *EIIa-SW* and *Col2-SW* mice at P7 and P21 (Fig. 2a and Supporting Table 5). The palatine process of maxilla was incompletely formed in *EIIa-SW* mice. Histological observation in the midsagittal plane of the facial region also showed the fusion between the premaxilla and the vomer in *EIIa-SW* and *Col2-SW* mice (Fig. 2b). No cartilaginous tissue was found between the two bones. The snout was crooked in both sets of mutant mice (Fig. 2c). Furthermore, the formation of the perpendicular plate of the ethmoid bone was significantly reduced in both mutant groups (Fig. 2d,e). Interestingly, the affected facial bone components in mutant mice are adjacent to the septal cartilage in the center. Therefore, the growth changes of septum may cause these midface deformities of the mutant mice.

### Thickening of septal cartilage altered the paraseptal structures in mutant mice

**Figure 3:**
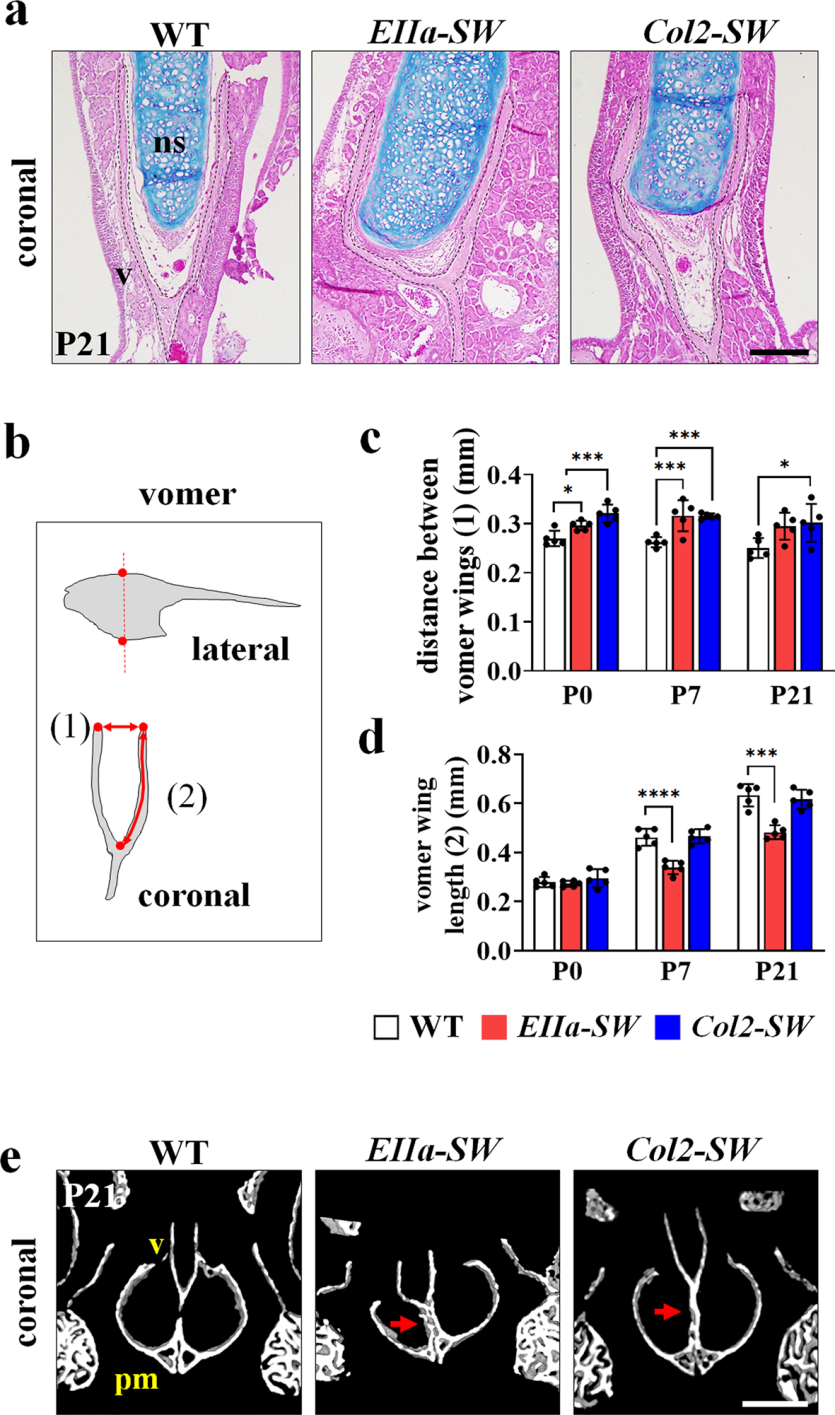
Thickening of septal cartilage altered the paraseptal structures in mutant mice. (**a**) Hematoxylin, eosin, and Alcian blue staining of the septal cartilage (ns) and vomer (v) at P21. The dotted line indicates the vomer. Scale bar: 200 µm. (**b**) Schematic image of the vomer for linear measurements. The measurements were conducted in the coronal plane of 3D reconstructed images, at the highest and the lowest points on the vomer are seen (red dotted line). The distance between the two vomer wings (**c**) (distance (1) in Fig. 3b) and the length of the vomer wings (**d**) (length (2) in Fig. 3b) were measured. The lengths of the two wings were averaged (n = 5). (**e**) Micro-CT images of the vomer and premaxilla bone in the coronal plane. The red arrows indicate the fusion between the vomer (v) and the premaxilla bone (pm). Scale bar: 0.5 mm. Values are presented as means ± standard deviations. *p ≤ 0.0332, **p ≤ 0.0021, ***p ≤ 0.0002, ****p ≤ 0.0001.

Anatomically, the inferior part of the septal cartilage is flanked by Y-shaped vomer wings in the coronal view (Fig. 3a). We found that the septal cartilage in *EIIa-SW* and *Col2-SW* mice was dramatically thickened. This structural change in the septum caused widening and distortion of the surrounding vomer wings (Fig. 3a–c). The vomer wing length was reduced in *EIIa-SW* mice but not in *Col2-SW* mice (Fig. 3d). As shown in Fig. 2a, vomero-premaxillary suture disappeared, and the vomer shape was distorted in the mutant mice in the mid-coronal plane (Fig. 3e). These histological observations suggest that the thickening of septal cartilage may result in passive displacement of vomer and the related facial bone structures in the mutant mice.

### Thickening and deviation of nasal septal cartilage caused crooked snout in mutant mice

**Figure 4:**
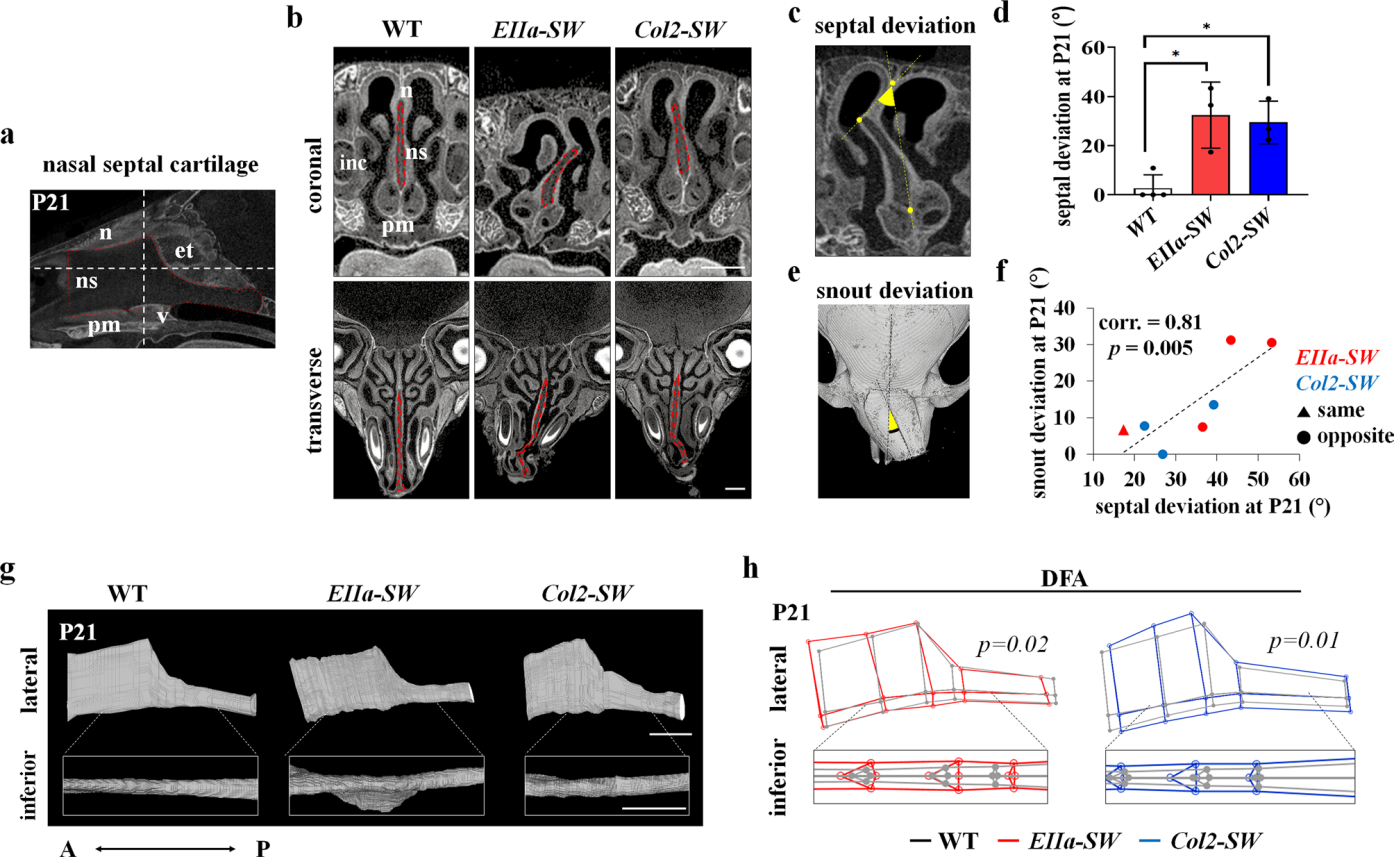
Thickening and deviation of nasal septal cartilage caused crooked snout in mutant mice. (**a**) Midsagittal micro-CT image of the nasal cavity of mice at P21, which were stained with potassium triiodide, the contrast agent (Lugol solution), for 48 h. The dotted red line indicates the nasal septum. The white line indicates the coronal and transverse planes for further analyses. Abbreviations: n, nasal bone; ns, nasal septum; pm, premaxilla; v, vomer; et, the perpendicular plate of the ethmoid bone. (**b**) Coronal view (upper images) and transverse view (lower images) of the nasal cavity. The dotted red line indicates the septal cartilage. Scale bar: 1 mm. (**c**,**d**) The measurement of septal deviation in the coronal view of the nasal cavity stained with the contrast agent. The measured plane was matched between groups based on the structural features of incisors, premaxilla, eyeballs and vomeronasal organ. The angle of septal deviation between the most deviated point of the septum and the midline (crossing two points on the nasal bone and the caudal most point of the septum) was measured (n ≥ 3). (**e**) Measurement of nasal deviation. The angle between the two dotted black lines, parallel to the interfrontal suture and the nasal bone, was measured (n ≥ 3). (**f**) Correlation between the degree of deviation of the snout and the septum in the mutant mice. The triangle indicates the same deviating direction of the nasal septal cartilage and nose, and the circle indicates the direction opposite of deviation. (**g**) 3D reconstructed images of the nasal septal cartilage at P21 in lateral and inferior views (magnified in the white box). Scale bar: 1 mm. (**h**) Mean septal cartilage shape of *EIIa-SW* and *Col2-SW* was compared with that of WT mice by DFA with the wireframe images. The inferior view of wireframe image is displayed in the box (*n* ≥ 3). The *p* values for 1000 permutation tests between two groups are shown with the wireframe images. Values are presented as means ± standard deviations. *p ≤ 0.0332.

We stained the samples with contrast enhancing agent to examine the morphological changes of the nasal septal cartilage. In the midsagittal plane, the levels of coronal section and transverse section shown in Fig. 4b were depicted with dotted lines in Fig. 4a. On the coronal plane in the middle of the septal cartilage, *EIIa-SW* and *Col2-SW* mice displayed thickened and deviated septal cartilage (Fig. 4b, upper images, and Fig. 4c,d). *EIIa-SW* mice showed more severe deviation with lowered nasal bone. The major point of the deviation was at the junction between the nasal bone and the septal cartilage. The deviation of the snout was observed in the transverse plane as well (Fig. 4a,b, lower images). The degrees of nasal and septal deviation were significantly correlated (Fig. 4e,f). Six of seven mutant mice showed nasal deviation on the opposite side of nasal septum deviation (Fig. 4f). On the 3D-reconstructed images, and according to a comparison of the mean shape of septal cartilage, the anteroposterior length was reduced, and thickening and morphological changes in height were evident in both mutant groups (Fig. 4g,h). These notable morphological changes already have been seen at earlier stage (Supporting Fig. 8). These results demonstrated that the chondrocyte-specific *Fgfr2*^*S252W*^ mutation induces thickening of septal cartilage that is sufficient to cause the septal deviation and facial deformities.

### Increased chondrocyte hypertrophy may cause thickening of the nasal septum

**Figure 5:**
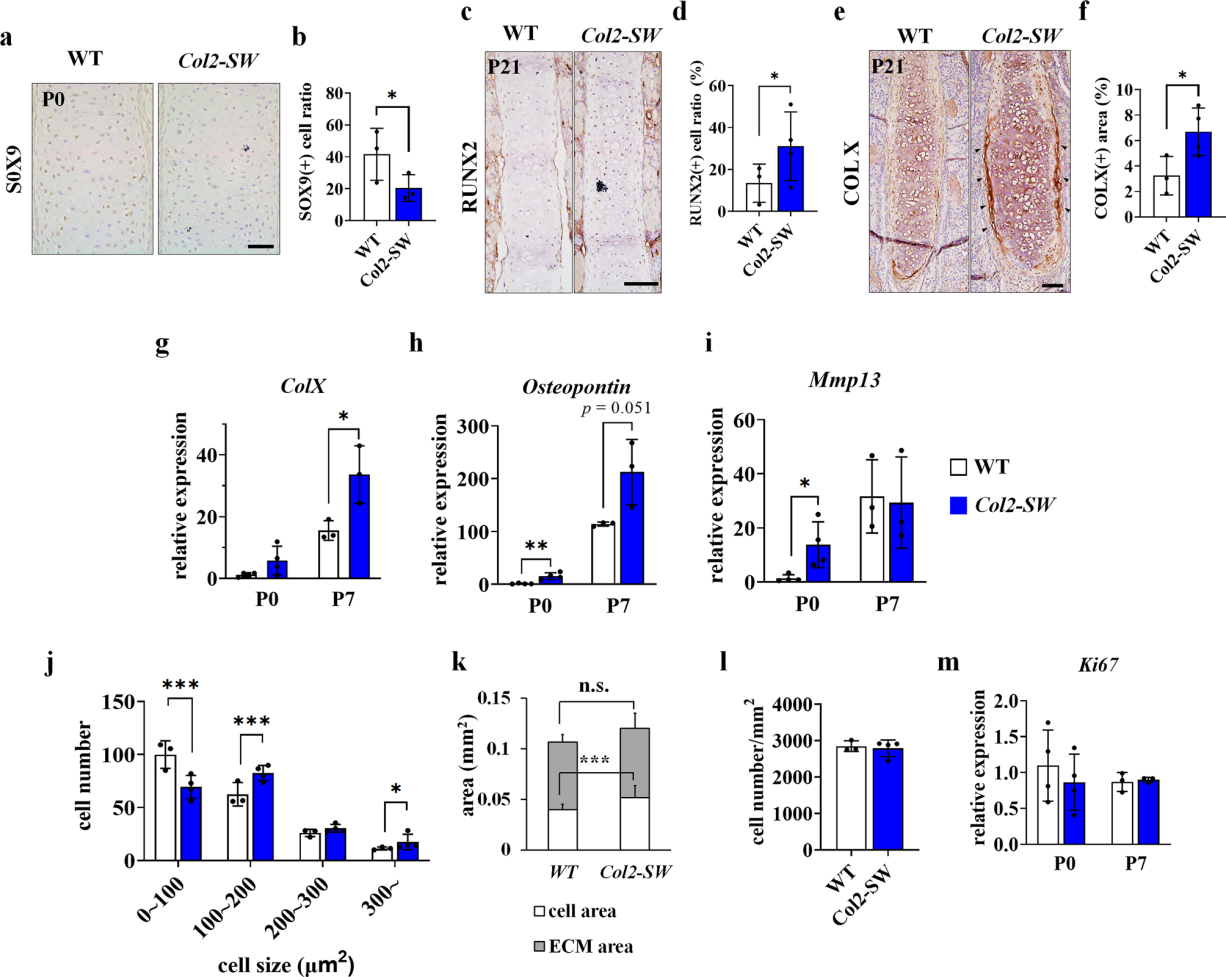
Increased chondrocyte hypertrophy and cellular size, which induced thickening of the nasal septal cartilage. (**a**, **b**) Immunohistochemistry of SOX9 and SOX9-positive cell ratio in the septal cartilage at P0. Scale bar: 50 µm. Three mice per genotype were used. (**c**,**d**) Immunohistochemistry of RUNX2 and RUNX2-positive cell ratio in the septal cartilage of mice at P21. Scale bar: 100 µm. (**e**,**f**). Immunohistochemistry of type X collagen (COLX) and COLX-positive area ratio in the septal cartilage of mice at P21. Positive cell and area ratio from randomly selected three slides from each mouse was averaged. The black arrowheads indicate the expression of COL X at the edge of the septal cartilage of *Col2-SW*. Scale bar: 100 µm. For RUNX2 and COLX positive cell or area measurements, three WT mice and four *Col2-SW* mice were used. Three slides were randomly selected from each mouse. Gene expression of markers of chondrocyte hypertrophy: *Col10a1* (**g**), *Osteopontin* (**h**), and matrix metalloproteinase-13 (*Mmp13*) (**i**) in the septal cartilage at P0 and P7. The expression level was normalized to *Gapdh*. RNA from P0 mice (n = 4 per genotype) and P7 mice (n = 3 per genotype) were used. (**j**) Septal chondrocyte size distribution in the septal cartilage at P21. (**k**) The extracellular matrix (ECM) (gray) and cellular (white) area in the septal cartilage at P21. The difference of ECM area between two groups was not significant. (**l**) The cell density (cell number/area) was measured in the septal cartilage at P21. For histological assessments (**j**–**l**), three WT mice and four *Col2-SW* mice were used. Four slides were randomly selected from each mouse. (**m**) Gene expression of *Ki-67* in the septal cartilage at P0 and P7. Values are presented as means ± standard deviations. For Student’s *t* test, *p ≤ 0.05, **p ≤ 0.01, ***p ≤ 0.001. n.s., not significant.

To examine the effect of *Fgfr2*^*S252W*^ on the septal chondrocyte, we conducted gene expression and histological assessments of the septal cartilage of *Col2-SW* mice. Expression levels of SOX9, a marker of early-stage chondrocytes, were decreased at P0 stage (Fig. 5a,b). By contrast, RUNX2, a marker of hypertrophic chondrocytes, were increased in the mutant septal cartilage on P21 (Fig. 5c,d). The gene and protein expression of type X collagen were also increased on the septal cartilage matrix of *Col2-SW*, which indicated an increase in chondrocyte hypertrophy and changes in septal matrix structure (Fig. 5e–g). The expression of other hypertrophy markers, *Osteopontin* and matrix metalloproteinase-13 (*Mmp13*), were also increased in the mutant septal chondrocytes (Fig. 5h,i). These changes at early stage caused significant morphological differences even at P7 stage (Supporting Fig. 8).

As cellular hypertrophy accelerated, the cellular size of septal chondrocytes was also significantly increased (Fig. 5j). The cellular area occupying the septal cartilage was increased by 5.1% (Fig. 5k). While, the extracellular matrix area was not significantly changed, indicating that the increased cellular area might be the primary cause of the septal cartilage thickening. However, the density of septal chondrocytes and proliferation were not significantly different (Fig. 5l,m). As septal cartilage area increased, cell density was not decreased even with the increased cell size. Collectively, *Fgfr2*^*S252W*^ mutations on the nasal septal chondrocyte increased cellular hypertrophy, and altered the nature of the cartilage matrix.

## Discussion

In patients with Apert syndrome, midface hypoplasia and airway obstruction are the major problems that must be managed during childhood^6^. However, the pathogenic mechanism and the intrinsic contributing factors of midface hypoplasia remain unclear. Breathing problems caused by a deviated nasal septum, choanal atresia, and narrowed tracheal cartilage sleeve^9^ are related to abnormalities in the nasal septum or facial cartilaginous tissue growth. In this study, we demonstrated that abnormal septal cartilage growth may act as a major contributor to midface hypoplasia in a mouse model.

Patients with Apert syndrome are treated with decompressive surgery at early stage, since the cranium grows rapidly during the first 5 years^13^. However, midface hypoplasia should be treated and managed for long periods, since the maxillary growth is still active at the ages of 12 to 14^14^. Hypoplastic facial phenotypes of *Col2-SW* mice in the absence of premature craniofacial suture closure also imply the importance of intrafacial features in Apert syndrome. The premature closure of the vomero-premaxillary suture found in *Col2-SW* mice could cause the anteroposterior facial shortening and deformities^15,16^. The distortion of the vomer could also result in respiratory failure^17^, however, this abnormal fusion was not expected because the premaxilla and the vomer do not grow by endochondral bone formation^14^. In the mutant mice, we also found elevated nasion, deviated nasal bones, and reduced formation of the perpendicular plate of the ethmoid bone^18^. These phenotypes strongly indicate the involvement of changes in septal cartilage growth in abnormal midfacial phenotypes. Increased chondrocyte hypertrophy and cellular size, altered septal matrix structure, and enlarged septal cartilage would impinge upon the vomer and nasal bone, resulting in facial deformities^19,20^.

The abnormal fusion between the premaxilla and the vomer in *Col2-SW* mice can be explained with the secondary effect from abnormal thickening of septal cartilage above, however, there still is a possibility that the expression of Col2a1-cre in the skeletal lineage cells and suture mesenchyme between two bones^21^. The expression of Fgfr2^S252^^W^ mutation in the suture mesenchyme could directly effect on the abnormal fusion. Close examination for Col2a1-cre expression profile in the facial region should be studied further.

For the patients with Apert syndrome, immediate surgical treatment of the cranium should be conducted early in the patient’s life to relieve severe respiratory distress and intracranial hypertension^22^. Later during growth and even up to adulthood, serial anterior skull and facial advancement are considered as a second step. Similar to humans with Apert syndrome, *EIIa-SW* mice with craniosynostosis showed severe craniofacial anomalies at birth^23,24^. By contrast, *Col2-SW* mice showed normal craniofacial phenotypes at birth and relatively progressive development of midface hypoplasia, especially with abnormally thickened septal cartilage. Therefore, surgical resection of thickened septum during the treatment of Apert syndrome may improve midfacial development and relieve the burden of multiple surgical steps.

The septal overgrowth might have resulted in the septal deviation^20^. Two mutant mice displayed septal deviation, however, *EIIa-SW* mice showed more severe septal deviation because of the premature closure of the nasofrontal suture and the sunken nasion, on the other hand, *Col2-SW* showed normal suture development and elevated nasion. These phenotypes may indicate that the vertical thickening of septal cartilage and premature facial suture fusion influence each other causing septal deviation and facial deformities in the mutant mice. The deviation occurred mainly between the nasal bone and septal cartilage, and it reflected the hardened matrix structure of septal cartilage.

Septal deviation could also result in the abnormal facial growth in relation with vomer and premaxilla development^25,26^. Although the fusion of vomero-premaxilla suture could effect the snout deviation^27^, we found that the degree of septal deviation and snout deviation were strongly correlated^28^. Furthermore, horizontal sectional views showed that the snout deviation occurred to the opposite side of the septal deviation in most of the mutant mice. Likewise, in human patients with septal deviation, an external anteroposterior C-shaped septal deviation is commonly on the side opposite to the internal deviation^29^. However, because humans and mice have structural differences, closer investigation is needed. The data in this study imply that septal cartilage has an essential role in determining the facial shape; furthermore, the application of surgical septal correction in the treatment of midface hypoplasia could enable proper facial growth.

The facial deformities and the morphological changes in a mouse model of Apert syndrome have been well studied. Prenatal craniofacial suture closure induced mainly decreased antero-posterior growth and a relative increase in width and height^30,31^. These growth patterns were also observed during the postnatal growth in our study, and the anomalies and the size-related differences became more severe as the mice grew. However, most previous studies have addressed mainly early craniofacial suture closure as a major cause of the deformities^30,32,33^. Holmes et al. first reported the abnormal growth of nasal cartilaginous tissues and the possible effect on the midface dysgenesis at prenatal and neonatal stages^34^. In this study, we have suggested the nasal septum overgrowth as another major causal factor in midface hypoplasia, and we performed assessment of morphological changes and growth pattern at postnatal stages. Our findings may provide the new insight into management of the facial anomalies of patients with Apert syndrome.

FGF signaling accelerates hypertrophic differentiation and reduces its proliferation mainly through FGFR3 in the differentiation of chondrocyte^35^. Some investigators have studied the cartilage issues found with the *Fgfr2* activating mutations as well. In syndromic craniosynostosis, including Apert syndrome, solid tracheal cartilage sleeve is commonly reported as a respiratory defect in humans and mouse models^36,37^. A recent study also demonstrated thickened nasal septum and narrowed nasal passages in the mouse model of Apert syndrome at the neonatal stage^34^. Similarly, our data demonstrated increased septal chondrocyte hypertrophy and thickening of the septum. Furthermore, 3D septal shape analysis and histological assessments during growth highlighted the importance of septal growth in the facial development and airway formation.

We confirmed that septal chondrocytes undergo hypertrophy during development. Other studies also showed the capacity of nasal septal chondrocytes for hypertrophy and mineralization under certain conditions in both in vitro and in vivo models^18,38^. *Fgfr2*^*S252W*^ mutations seem to accelerate this process in the nasal septum; however, we did not observe increased type I collagen expression, ectopic ALP activity or ossification at P21. The septum development at later stages of mutant mice should be observed in the further study. We also found the septal thickening is caused mainly by enlarged chondrocyte size rather than by an increase in the number of chondrocytes. Our previous observation also showed no significant change in chondrocyte proliferation in the cranial base synchondroses in the same mouse model^12^. By contrast, Holmes et al. has reported a transient increase of chondrocyte proliferation at the embryonic stage, but they also confirmed the thickening of septal cartilage without proliferation change at later stages^34^. These studies indicate that FGF signaling has a tissue- and stage-specific role in craniofacial cartilage development.

In this study, by using a mouse model with chondrocyte-specific *Fgfr2*^*S252W*^ expression, we demonstrated abnormal growth of nasal septal cartilage may be caused by increased cellular hypertrophy and its possible effects on the adjacent structures. Our detailed morphological analyses indicate that altered septal growth may critically contribute to the nasal obstruction and the midface deformities in the patients with Apert syndrome.

### Experimental procedures

#### Ethics statement

All methods were carried out in accordance with relevant guidelines and regulations. The animal experiments were carried out in compliance with the appropriate Animal Research: Reporting In Vivo Experiments (ARRIVE) guidelines. All mice were maintained under specific pathogen-free conditions in individual ventilating systems. Light was adjusted to a 12 h/12 h light/dark cycle. Temperature and humidity were regulated to 22 °C ± 3 °C and 50% ± 10%, respectively. Gamma-irradiated food and UV reverse osmosis water were provided on and ad libitum basis. All experimental protocols were approved by the Institutional Animal Care and Use Committee and Special Committee on Animal Welfare of Seoul National University (Seoul, Korea) (SNU-200923–2).

### Generation of tissue-specific mutant mice

A mouse carrying ploxPneo cassette, which blocks expression of the mutant *Fgfr2* allele (*Fgfr2*^*neoS252W/*+^, genetic background is approximately 88% FVB, 6% Black Swiss, and 6% 129SEVE)^39^, was bred with different types of Cre recombinase-expressing mice: *EIIa-cre* (B6.FVB-TgN [EIIa-cre] C3739Lm, 003,724; Jackson Laboratory, Bar Harbor, ME), *Col2a1-cre* (C57BL/6 X SJL)^40^, and *Col1a1*(2.3 kb)*-cre* (B6D2F1)^41^ mouse. *Col2a1-cre* and *Col1a1-cre* transgenic mice were kindly provided by Dr. Je-Yong Choi (Kyungpook National University, Daegu, Korea)^42,43^. Homozygotic Cre transgenic mice were used in this study. Both sexes of pups carrying *Fgfr2*^*S252W*^ mutation were examined as neonate (P0), and at the age of 1 week (P7) and 3 weeks (P21). The total number of used mice is presented in Supporting Fig. 3. All the mutant mice we had were used for the experiments, and the wild-type (WT) mice were randomly selected from among littermates, including mutant mice. The numbers of mice for each assessment are described in each figure legend.

### Micro-computed tomographic scanning and landmark placing

Mice were euthanized with CO_2_ inhalation, and the dissected heads were fixed with 4% paraformaldehyde. To acquire micro-CT scans of the heads, we used the SKYSCAN 1272 (Bruker, North Billerica, MA) with a resolution of 10 or 20 µm/pixel (n = 5). The samples were stained with an iodine-based contrast-enhancing agent (Lugol solution [L6146-1L]; Sigma–Aldrich, St. Louis, MO, USA) for 48 h and then scanned at a resolution of 10 µm/pixel for nasal septal cartilage examination (n ≥ 3)^44^. The septal cartilage area was manually selected from multiple slices in the coronal view and reconstructed with the use of CT Analyser software (Bruker). Using Landmark Editor 3.0v (IDAV, University of California, Davis, CA), we recorded 23 landmarks on the craniofacial bones (Supporting Fig. 1 and Supporting Table 1)^12,45^; and 15 landmarks on the nasal septal cartilage (Supporting Fig. 2 and Supporting Table 2).

### Shape analyses and statistics

Linear and volumetric measurements of the three-dimensionally (3-D) reconstructed skull, vomer, perpendicular plate of the ethmoid bone and nasal septal cartilage were conducted with the use of the CT Analyzer (Bruker) and TRI/3D-BON (RATOC System Engineering Co., Tokyo, Japan). Landmark coordinates were processed into the Procrustes superimposition to scale the objects uniformly and were used for principal components analysis and discriminant function analysis by MorphoJ v1.06d (Klingenberg Lab, Manchester, UK)^46^ as previously described^12^. To analyze the shape distribution among groups, we performed the Procrustes analysis of variance with the permutation test against the null hypothesis of independence with 1000 rounds of number of randomization. The septal deviation was measured on the coronal plane of the nasal cavity through the use of ImageJ software (National Institutes of Health, Bethesda, MD), (n ≥ 3). Values were calculated as means ± standard deviations, and Prism 8.4.3 (GraphPad Software, San Diego, CA) was used to perform with one-way analysis of variance, followed by the Tukey or Bonferroni multiple comparison test.

### Histological analysis

The specimens were processed as previously described^12^. The paraffinized dissected heads of the mice were serially sectioned into 5 µm-thick slices in the sagittal or coronal plane with a rotary microtome (RM2145; Leica, Wetzlar, Germany). Slides were stained with Alcian blue, hematoxylin, and eosin. To quantify the number and the size of cells in the septal cartilage, each chondrocyte was selected manually through the use of Osteomeasure software (OsteoMetrics, North Decatur, GA) and calculated with ImageJ software. For immunohistochemistry, SOX9 antibody (sc-20095; Santa Cruz Biotechnology, Dallas, TX), RUNX2 antibody (D130-3; MBL International Corporation, Woburn, MA), and collagen type X rabbit antibody (234,196, MilliporeSigma, Burlington, MA) were used after antigen retrieval. ImageJ software was used to count stained cells and positive area. More than three mice per genotype were used for the histological analyses. Positive cell or area ratio from randomly selected three slides from each mouse was averaged. For alkaline phosphatase (ALP) staining, an ALP stain kit (MK300, Takara Bio, Kyoto, Japan) was used in accordance with the manufacturer’s instructions.

### RNA isolation from nasal septal cartilage and gene expression analysis

Nasal septal cartilage was carefully isolated from mice after they were euthanized at P0 (n = 4) and P7 (n = 3). Soft tissues around the septal cartilage were cleaned with a scalpel, and the septal cartilage was directly placed into QIAzol Lysis Reagent (79,306; Qiagen, Hilden, Germany). Isolated RNA was synthesized into complementary DNA (RR036A; Takara Bio, Kyoto, Japan), and a quantitative real-time PCR was conducted with TB Green Premix Ex Taq TM (Tli RNaseH Plus (RR420A); Takara Bio, Kyoto, Japan). Supporting Table 3 shows the details of the primers.

## Supplementary Information

Supplementary Information.

## Data Availability

The data sets used and/or analyzed during the current study are available from the corresponding authors upon reasonable request.

## Abbreviations

*FGFR2*: Fibroblast growth factor receptor 2
*EIIa-SW*: *EIIa-cre; Fgfr2*^*S252W/*^^+^
*Col2a1-SW*: *Col2-cre; Fgfr2*^*S252W/*^^+^
*Col1-SW*: *Col1a1-cre; Fgfr2*^*S252W/*^^+^
*PCA*: Principal components analysis
*DFA*: Discriminant function analysis
*micro-CT*: Micro-computed tomography
*RUNX2*: Runt-related transcription factor 2
*ALP*: Alkaline phosphatase
