# Supplementary material for: Septal chondrocyte hypertrophy contributes to midface deformity in a mouse model of Apert syndrome
Source: Sci Rep. 2021 Apr 12;11:7979. doi: 10.1038/s41598-021-87260-5 (PMC8041873; doi:10.1038/s41598-021-87260-5)
Supplement: Supplementary file 1 — Supplementary Information. [file 41598_2021_87260_MOESM1_ESM.docx]

**Supporting information**

**Septal chondrocyte hypertrophy contributes to midface deformity in a mouse model of Apert syndrome**

Bong-Soo Kim^1^, Hye-Rim Shin^1^, Hyun-Jung Kim^1^, Hee-In Yoon^1^,

Young-Dan Cho^2^, Kang-Young Choi^3^, Je-Yong Choi^4^, Woo-Jin Kim^1^ and Hyun-Mo Ryoo^1^

^1^Department of Molecular Genetics and Dental Pharmacology, School of Dentistry and Dental Research Institute, Seoul National University, Seoul, South Korea

^2^Department of Periodontology, School of Dentistry and Dental Research Institute,

Seoul National University, Seoul, South Korea

^3^Department of Plastic and Reconstructive Surgery, School of Medicine,

Kyungpook National University, Daegu, South Korea

^4^Department of Biochemistry and Cell Biology, Cell and Matrix Research Institute, Skeletal Disease Analysis Center, Korea Mouse Phenotyping Center (KMPC), School of Medicine, Kyungpook National University, Daegu, South Korea


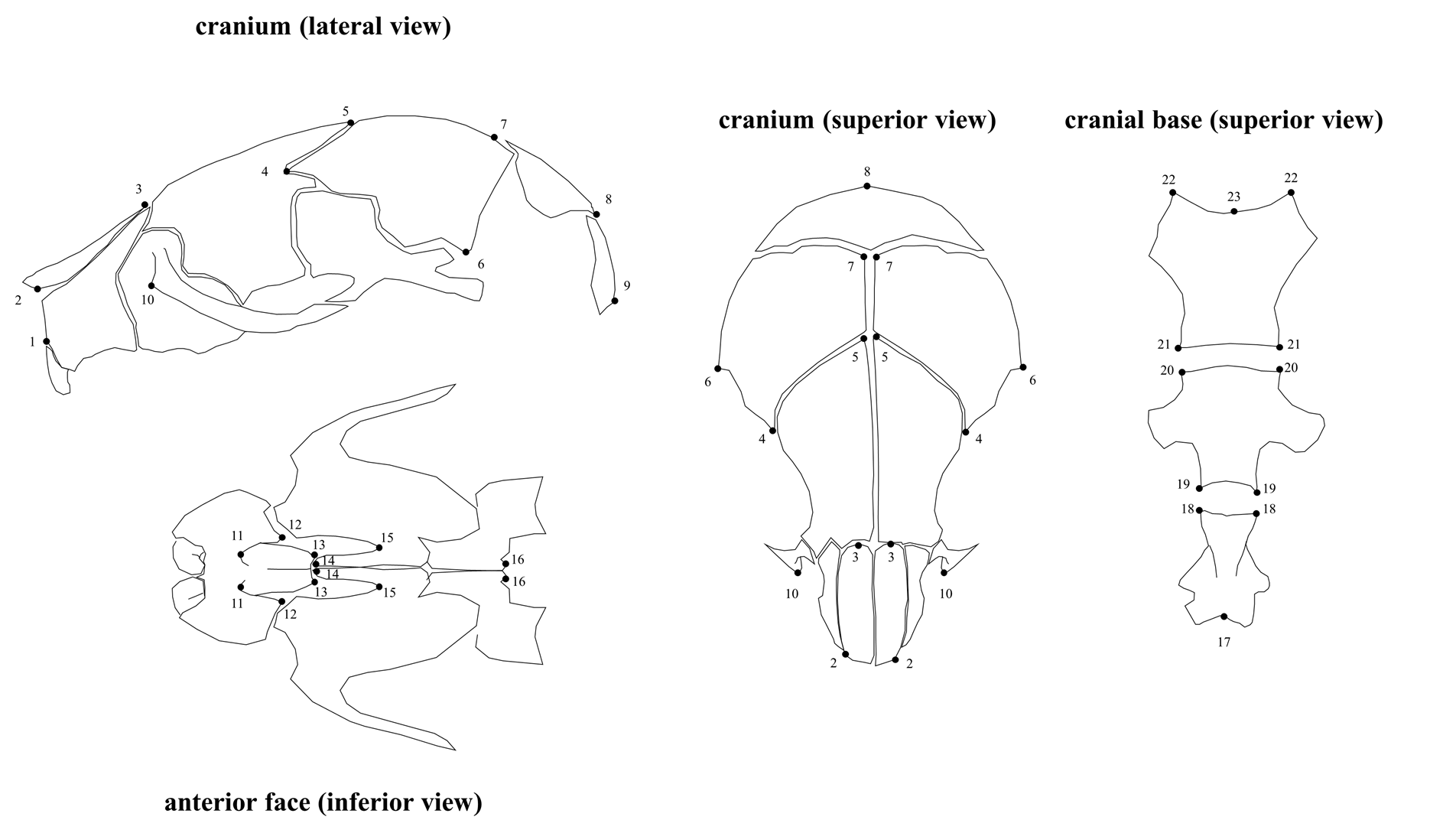


**Supporting Figure 1.** Craniofacial landmarks for morphometric analyses. Descriptions for the landmarks are provided in Supporting Table 1.


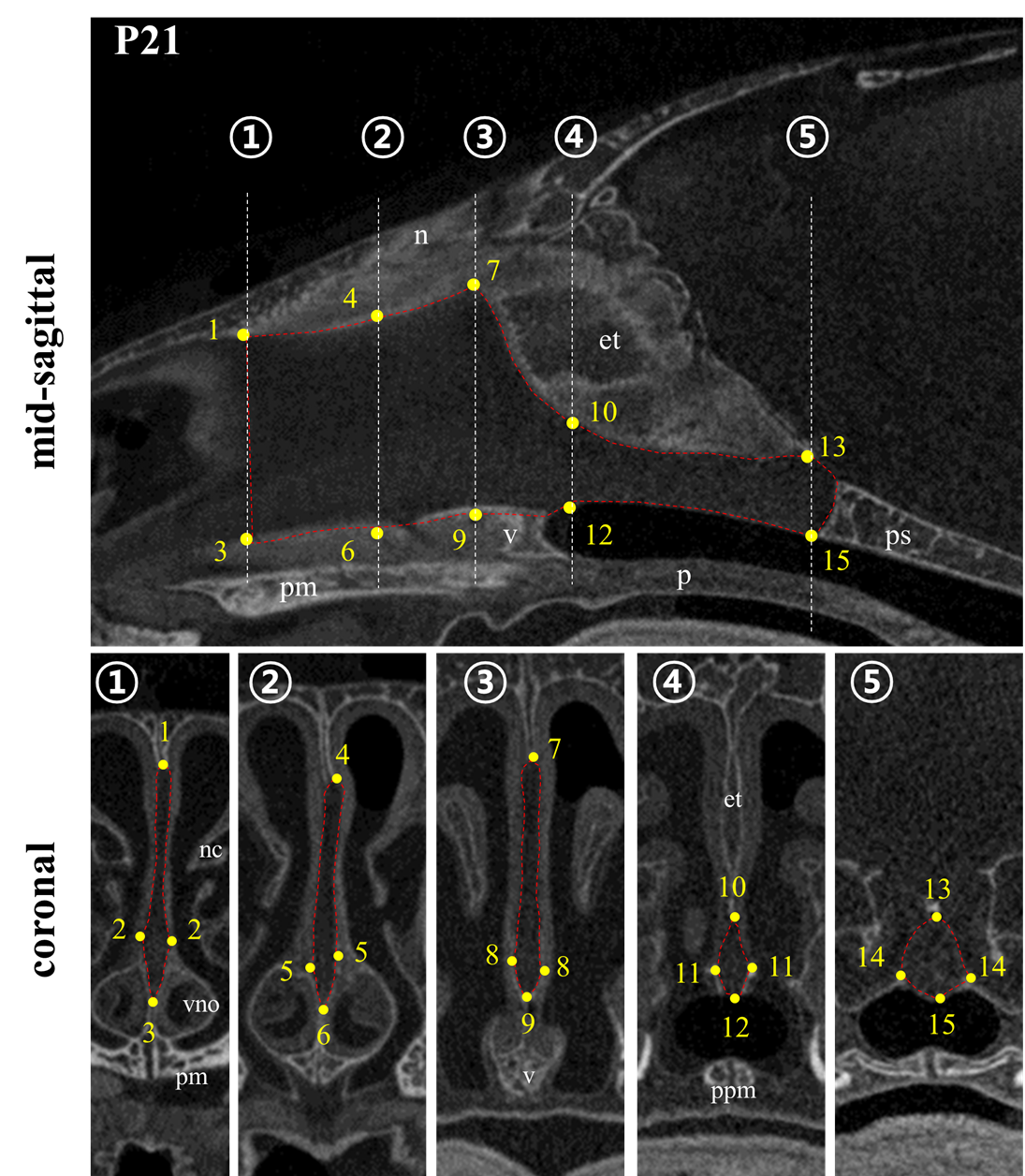


**Supporting Figure 2**. Landmarks on the nasal septal cartilage for 3D shape analysis. Red dotted area presents manually selected septal cartilage region. Plane ① was selected where the incisive foramen ends and two premaxilla plates meet. The position of all specimens was equally aligned considering the shape and size of premaxilla bone, nasal concha, incisor root and vomeronasal organ. Abbreviations: n, nasal bone; et, ethmoid bone; pm, premaxilla bone; v, vomer; p, palate; nc, nasal concha; vno, vomeronasal organ; ppm, process of palatine bone of maxilla. Plane ②, ③, ④ and ⑤ are parallel to Plane ① crossing the landmarks 4, 7, 10 and 13, respectively. Descriptions for landmarks are on Supporting Table 2.


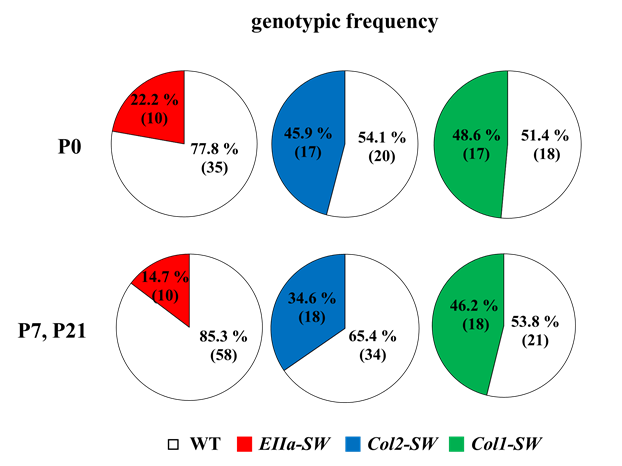


**Supporting Figure 3**. The genotypic frequency of each mutant mouse. The genotypes were checked on the day of delivery (P0), P7 or P21. The genotypic rate at P0 or P7, P21 stage was provided in the graph and the number of mice is in parentheses. The expected genotypic ratio of WT and *Fgfr2^S252W/+^* is 1:1 as *Fgfr2^neoS252W/+^* mouse was crossed with homozygous *Cre*-transgenic mouse. *EIIa-SW* showed neonatal lethality, and only 14.7 % of mutant mice survived after P7. *Col2-SW* showed normal birth rate, but postnatal lethality was seen as they grow. *Col1-SW* did not show lethality.


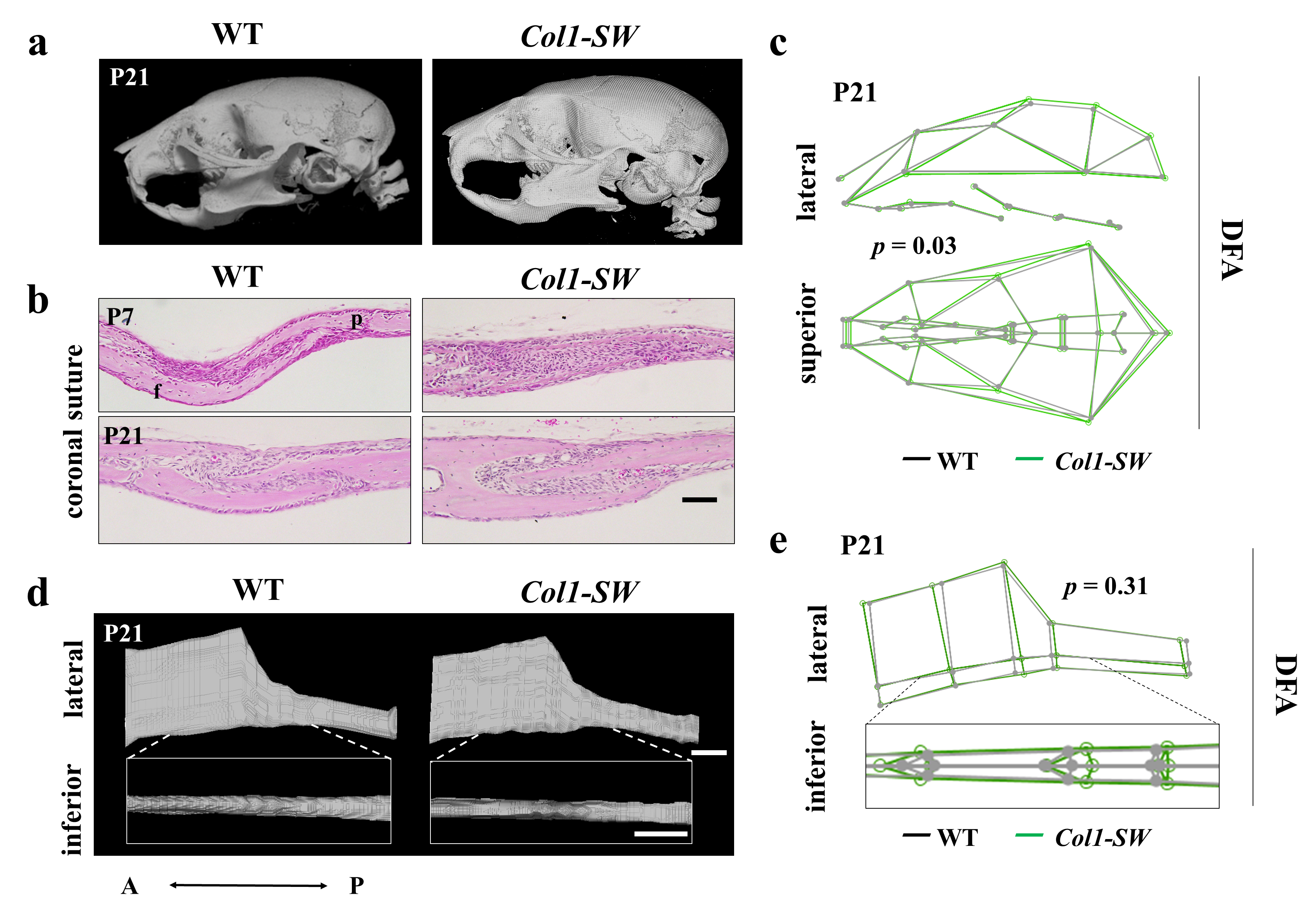


**Supporting Figure 4**. Morphometric analysis of *Col1-SW* mice.

**(a)** Micro-CT images of skull of WT and mature osteoblast-specific *Col1a1-cre; Fgfr2^S252W/+^* mice. Scale bar: 2 mm. **(b)** Histology of coronal suture at sagittal section. No suture fusion was found in *Col1a1-cre; Fgfr2^S252W/+^* mice. Scale bar: 100 μm. **(c)** The craniofacial shapes of two groups were compared in DFA. No noticeable morphological changes were found (n = 5). The p values for 1000 permutation tests between two group are shown with the wireframe images. **(d)** 3D reconstructed images of the nasal septal cartilage at P21 in lateral and inferior views (magnified in the white box). Scale bar: 1mm. **(e)** Mean septal cartilage shape of Col1-SW was compared with that of WT by DFA with wireframe images. The inferior view of wireframe image is displayed in the box (*n*≥ 3). The *p* values for 1000 permutation tests between two groups are shown with the wireframe images.


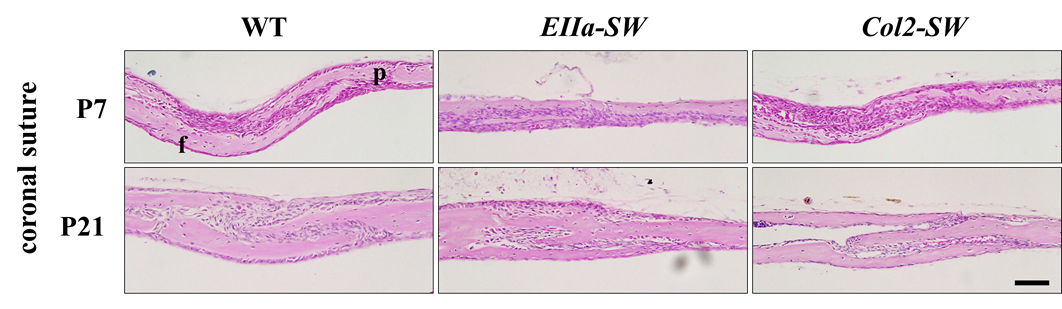


**Supporting Figure 5.** Histology of coronal suture of *Fgfr2^S252W/+^* mutant mice. Hematoxylin and eosin staining of coronal suture at P7 and P21 stages. Frontal bone (f) and parietal bone (p) were fused in *EIIa-SW*, not in *WT* and *Col2-SW*. Scale bar: 100 μm.

**Supporting Figure 6.** The linear measurements of facial and cranial regions (related to Figure 1c and f). Values are presented as means ± standard deviations. **p* ≤ 0.0332, ***p* ≤ 0.0021, ****p* ≤ 0.0002, *****p* ≤ 0.0001 (*n* = 5).


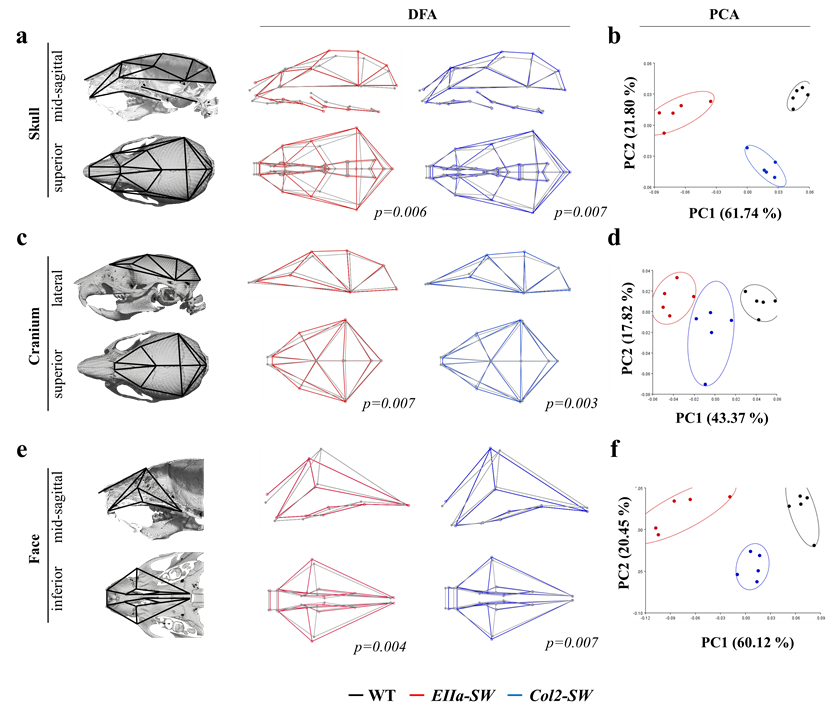


**Supporting Figure 7**. Morphometric analysis of whole skull, cranium and face. Based on the craniofacial landmarks (Supporting Fig. 1 and Supporting Table 1), 3D shape changes and patterns were analyzed. The mean shape of whole skull, the cranium and the facial bone is represented with wireframe images by discriminant function analysis (DFA) (a, c and e). *EIIa-SW* showed sunken nasion, elevated and widened cranium, bent cranial base and widened facial region. *Col2-SW* mouse showed elevated nasion and cranium and widened face. *p*-values for permutation tests (1000 permutations) between two groups are showed below of the wireframe images. The variance in the facial shape was analyzed by principal components analysis (PCA) (b, d and f).


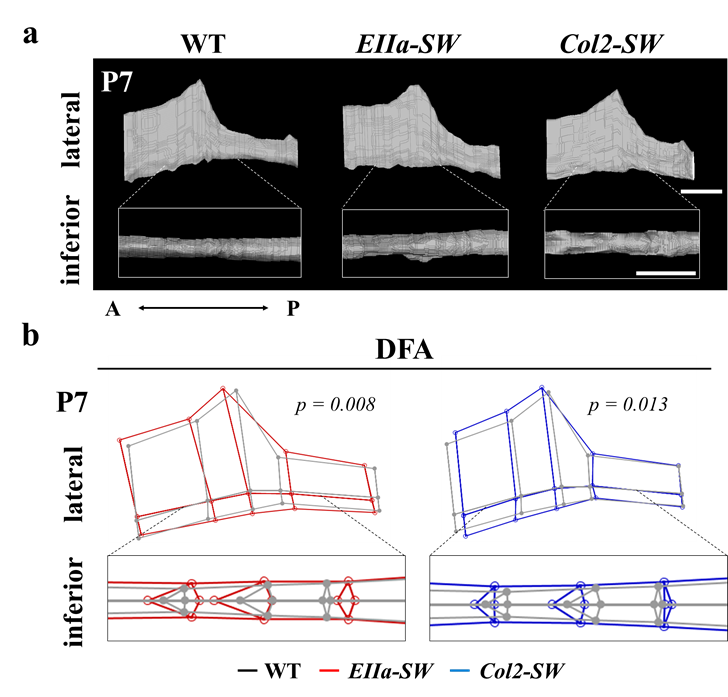


**Supporting Figure 8**. Morphometric analyses of septal cartilage at P7. **(a)** 3D reconstructed images of the nasal septal cartilage at P7 in lateral and inferior views (magnified in the white box). Scale bar: 1mm. **(b)** Mean septal cartilage shape of *EIIa-SW* and *Col2-SW* at P7 was compared with that of WT mice by DFA with the wireframe images. The inferior view of wireframe image is displayed in the box (*n*= 5). The *p* values for 1000 permutation tests between two groups are shown with the wireframe images.

**Supporting Table 1**. Landmark description used on the skull shape analysis

| **Landmark** | **Description** | **module** |
| --- | --- | --- |
| **1** | Point A, anterior-most point of alveolus of upper incisor (left and right) | face |
| **2** | Most antero-lateral point of corner of the nasal bone (left and right) | face |
| **3** | Nasion, caudal point of nasal bone (left and right) | face, cranium |
| **4** | Most superior point on the squamous temporal, intersection of the coronal suture (left and right) | cranium |
| **5** | Most medial intersection of the frontal and parietal bones, taken on the frontal (left and right) | cranium |
| **6** | Most postero-inferior point on the parietal bone (left and right) | cranium |
| **7** | Most postero-medial point on the parietal bone (left and right) | cranium |
| **8** | Caudal-most point of interparietal bone at mid-sagittal plane | cranium |
| **9** | Caudal-most point of occipital bone at mid-sagittal plane | cranium |
| **10** | Anterior notch on zygomatic process (left and right) | face |
| **11** | Most anterior point of the anterior palatine foramen (left and right) | face |
| **12** | Most infero-lateral point of the premaxillary-maxillary suture, taken on premaxilla (left and right) | face |
| **13** | Caudal-most point of premaxilla bone at mid-sagittal plane (left and right) | face |
| **14** | Most anterior point of palatine process of maxilla (left and right) | face |
| **15** | Most posterior point of the anterior palatine foramen (left and right) | face |
| **16** | Caudal-most point of palatine bone at mid-sagittal plane (left and right) | face |
| **17** | Rostral, dorsal-most point of presphenoid bone at mid-sagittal plane | cranial base |
| **18** | Most postero-lateral, dorsal point of corner of the presphenoid bone (left and right) | cranial base |
| **19** | Most antero-lateral, dorsal point of corner of the basisphenoid bone (left and right) | cranial base |
| **20** | Most postero-lateral, dorsal point of corner of the basisphenoid bone (left and right) | cranial base |
| **21** | Most antero-lateral, dorsal point of corner of the basioccipital bone (left and right) | cranial base |
| **22** | Most postero-lateral, dorsal point of corner the basioccipital bone (left and right) | cranial base |
| **23** | Basion, caudal, dorsal-most point of basioccipital bone at mid-sagittal plane | cranial base |

**Supporting Table 2**. Landmark description used on the nasal septal cartilage shape analysis

| **Landmark** | **Description** |
| --- | --- |
| **1** | Most superior point of septum at the plane ①^*^ |
| **2** | Most lateral point of septum at the plane ① |
| **3** | Most inferior point of septum at the plane ① |
| **4** | Most concave point of septum adjacent to the nasal bone. The plane ② is parallel to plane ① crossing landmark **4**. |
| **5** | Most lateral point of septum at the plane ② |
| **6** | Most inferior point of septum at the plane ② |
| **7** | The highest point of septum at the nasion. The plane ③ is parallel to plane ① crossing landmark **7**. |
| **8** | Most lateral point of septum at the plane ③ |
| **9** | Most inferior point of septum at the plane ③ |
| **10** | Most concave point of septum adjacent to the perpendicular plate of ethmoid bone. The plane ④ is parallel to plane ① crossing landmark **10**. |
| **11** | Most lateral point of septum at the plane ④ |
| **12** | Most inferior point of septum at the plane ④ |
| **13** | Most superior point of septum at the boundary of presphenoid bone. The plane ⑤ is parallel to plane ① crossing landmark **13**. |
| **14** | Most lateral point of septum at the plane ⑤ |
| **15** | Most inferior point of septum at the plane ⑤ |

* Description for the plane ① is described in Supporting Fig. 2

**Supporting Table 3**. Primers used for quantitative real-time PCR

| gene (mouse) | forward (5’ 🡪 3’) | reverse (3’ 🡪 5’) |
| --- | --- | --- |
| *Type X collagen* | TTCTGCTGCTAATGTTCTTGACC | GGGATGAAGTATTGTGTCTTGGG |
| *Osteopontin* | ATC TCA CCA TTC GGA TGA GTC T | TCA GTC CAT AAG CCA AGC TAT CA |
| *Mmp13* | CTT CTT CTT GTT GAG CTG GAC TC | CTG TGG AGG TCA CTG TAG ACT |
| *Ki-67* | CTG CCT GCG AAG AGA GCA TC | AGC TCC ACT TCG CCT TTT GG |
| *Gapdh* | CAT GTT CCA GTA TGA CTC CAC TC | GGC CTC ACC CCA TTT GAT GT |

**Supporting Table 4**. Craniofacial suture fusion frequency at P0 and P7 stages

|  |  | **craniofacial sutures** | | |
| --- | --- | --- | --- | --- |
| **age** | **genotypes** | **coronal** | **naso-frontal** | **premaxillo-maxilla** |
| **P0** | **WT** | 0/5 | 0/5 | 0/5 |
|  | ***EIIa-SW*** | 5/5 | 3/5 | 1/5 |
|  | ***Col2-SW*** | 0/5 | 0/5 | 0/5 |
| **P7** | **WT** | 0/5 | 0/5 | 0/5 |
|  | ***EIIa-SW*** | 4/5 | 5/5 | 5/5 |
|  | ***Col2-SW*** | 0/5 | 0/5 | 0/5 |

**Supporting Table 5**. Frequency of premaxilla-vomer closure at P7 and P21 stages

|  | **genotypes** | **premaxilla-vomer closure** |
| --- | --- | --- |
| **P7** | **WT** | 0/5 |
|  | ***EIIa-SW*** | 5/5 |
|  | ***Col2-SW*** | 5/5 |
| **P21** | **WT** | 0/5 |
|  | ***EIIa-SW*** | 5/5 |
|  | ***Col2-SW*** | 5/5 |
